# A systems-biology analysis of isogenic megakaryocytic and granulocytic cultures identifies new molecular components of megakaryocytic apoptosis

**DOI:** 10.1186/1471-2164-8-384

**Published:** 2007-10-22

**Authors:** Chi Chen, Peter G Fuhrken, Li Ting Huang, Pani Apostolidis, Min Wang, Carlos J Paredes, William M Miller, Eleftherios T Papoutsakis

**Affiliations:** 1 Interdepartmental Biological Sciences Program, Northwestern University, Evanston, IL, USA; 2 Department of Chemical and Biological Engineering, Northwestern University, Evanston, IL, USA; 3 Robert H. Lurie Comprehensive Cancer Center, Northwestern University, Chicago, IL, USA; 4 Dept of Chemical Engineering, Delaware Biotechnology Institute, University of Delaware, 15 Innovation Way, Newark, DE 19711, USA

## Abstract

**Background:**

The differentiation of hematopoietic stem cells into platelet-forming megakaryocytes is of fundamental importance to hemostasis. Constitutive apoptosis is an integral, yet poorly understood, facet of megakaryocytic (Mk) differentiation. Understanding Mk apoptosis could lead to advances in the treatment of Mk and platelet disorders.

**Results:**

We used a Gene-ontology-driven microarray-based transcriptional analysis coupled with protein-level and activity assays to identify genes and pathways involved in Mk apoptosis. Peripheral blood CD34^+ ^hematopoietic progenitor cells were induced to either Mk differentiation or, as a negative control without observable apoptosis, granulocytic differentiation. Temporal gene-expression data were analyzed by a combination of intra- and inter-culture comparisons in order to identify Mk-associated genes. This novel approach was first applied to a curated set of general Mk-related genes in order to assess their dynamic transcriptional regulation. When applied to all apoptosis associated genes, it revealed a decrease in NF-κB signaling, which was explored using phosphorylation assays for IκBα and p65 (RELA). Up-regulation was noted among several pro-apoptotic genes not previously associated with Mk apoptosis such as components of the p53 regulon and TNF signaling. Protein-level analyses probed the involvement of the p53-regulated GADD45A, and the apoptosis signal-regulating kinase 1 (ASK1). Down-regulation of anti-apoptotic genes, including several of the Bcl-2 family, was also detected.

**Conclusion:**

Our comparative approach to analyzing dynamic large-scale transcriptional data, which was validated using a known set of Mk genes, robustly identified candidate Mk apoptosis genes. This led to novel insights into the molecular mechanisms regulating apoptosis in Mk cells.

## Background

One hundred years after the identification of megakaryocytic (Mk) cells as the origin of platelets [1] consecutive yet distinct developmental stages and the associated stage-specific markers of megakaryopoiesis have now been well established. However, the molecular and cellular mechanisms through which these cells differentiate and mature remain poorly understood. Mk cells derive from bi-potent erythro-Mk progenitors [2,3]. Committed Mk progenitors undergo endomitosis and become polyploid with multilobated nuclei. At this stage, Mk cells undergo morphological changes including the development of a demarcation membrane system and dramatic increase in cell size [4]. Polyploidization and platelet release are linked to a program of constitutive apoptosis. While some reports have suggested that Mk apoptosis does not occur until after full maturation [5,6], a growing number suggest that apoptosis and maturation are intimately linked [7-11]. The peak in apoptotic cells during culture coincides with the peak in polyploidization [7,10]. The addition of caspase inhibitors delays proplatelet formation and postpones the peak (but not the onset) of polyploidization [10,11]. Although the mechanism by which Mk apoptosis is controlled is poorly understood, some evidence exists that the classical modulators of apoptosis are involved. *Bcl-x*_*L*_, an anti-apoptotic gene of the Bcl family, is up-regulated as Mk cells mature and then partitions to the shedding platelets [6]. *Bcl-x*_*L *_over-expression *in vivo *impairs recovery from immune thrombocytopenia and yields a small increase in Mk cell numbers [12]. Expression of *Bcl-2*, another anti-apoptotic gene, was decreased in a megakaryoblastic cell line and was low and unchanged during thrombopoietin (Tpo)-driven maturation of cord-blood CD34^+ ^cell derived Mk cells [6]. Bcl-2 over-expression throughout the murine hematopoietic compartment led to a 50% reduction in platelet levels with no change in Mk cell numbers [13]. Furthermore, irregular patterns of Mk apoptosis have been associated with Mk-cell-related diseases including immune thrombocytopenic purpura [14].

DNA-microarray-based genomic approaches have the potential to identify novel genes related to Mk differentiation and potentially link them to the existing knowledge base. Prior microarray studies have primarily examined Mk gene expression by comparing normal- and disease-state Mk cells [15], and by comparing culture-derived Mk cells to uncultured progenitor cells [16] or non-Mk cells [17] at single time points. Two recent papers have explored progressions of Mk differentiation using microarrays including murine Mks sorted by light-scattering properties and CD41 expression [18], and human Mks sorted by ploidy class [19]. Furthermore, two recent studies from our lab have exploited the richness of information from temporal analysis of Mk gene expression in both primary CD34^+ ^cell [20] and megakaryoblastic cell line cultures [21]. However, microarray data have not been systematically used to examine the transcriptional dynamics of important cellular programs related to megakaryopoiesis such as apoptosis, cell cycle, or cytoskeletal biology.

The goal of this study is to utilize global gene expression profiling and functional Gene Ontology (GO) classifications to identify Mk genes involved in apoptosis – a program of paramount importance to Mk differentiation. The GO classification allows one to select a set of genes of general or specialized cellular function or role that can be examined for gene expression patterns [22]. Previously, we utilized GO classifications to assess the underlying functional make-up of clusters formed by analysis of the raw data alone [21]. Here, we show that GO classifications can be used to successfully identify the genes involved in the Mk apoptosis program using the data from our high-quality DNA-microarray analysis without introducing any other selection biases.

## Results

### *Ex vivo *Mk differentiation of human CD34+ cells

**Figure 1:**
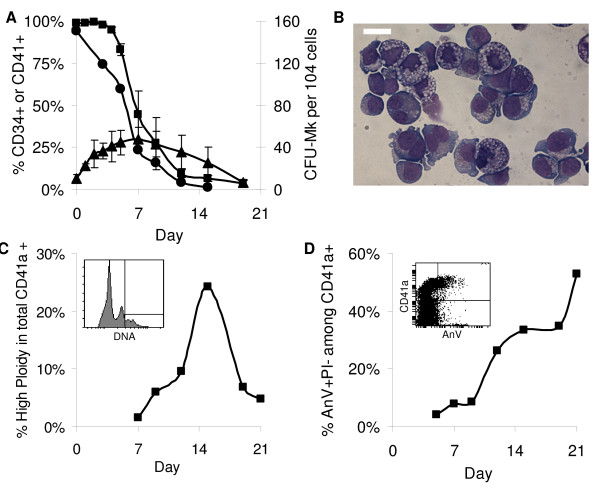
**Phenotypic analysis of *ex vivo *Mk differentiation from CD34^+ ^progenitor cells**. Mobilized peripheral blood CD34^+ ^cells were cultured in serum free media with an Mk-inducing cytokine cocktail of IL-3, Flt3-L and Tpo. (A) Percentages of viable cells expressing CD34 (squares) and CD41a (triangles) in Mk cultures, as assessed by flow cytometry (n = 3). The decline in Mk progenitor cell frequency, as measured by the CFU-Mk assay in a representative experiment (circles), slightly preceded loss of CD34 expression. (B) Wright-Giemsa stained cells from day 7 Mk culture displaying Mk cell morphological features. Image acquired with 63× objective shows representative field. Scale bar represents 20 μm. (C) The percentage of high ploidy (≥ 8N) among CD41a^+ ^cells from one representative experiment (n = 3). Inset shows example DNA histogram and gating from day 12. (D) The percentage of apoptotic (Annexin V^+^/PI^-^) cells among CD41a^+ ^cells from one representative experiment (n = 3). Inset shows example scatter plot and gating from day 11.

CD34^+ ^stem and progenitor cells from three healthy donors were cultured for 21 days with Tpo, interleukin (IL)-3 and Flt-3-ligand, a cytokine combination that promotes moderate expansion and differentiation along the Mk lineage [9]. The number of total nucleated cells continuously increased over time and reached 54(± 9)-fold by day 19 (mean ± SEM, n = 3), whereas the total Mk production (CD41a^+ ^cells per input CD34^+ ^cell) peaked at 7.5 between days 9 and 12 (data not shown). As early as day 2, 21 ± 8% of the cells in culture expressed CD41a (Figure 1A). The percentage of CD41a^+ ^cells increased until day 9 as the percentage of CD34^+ ^cells decreased (Figure 1A). The number of Mk progenitor cells, as measured by Mk colony formation in semi-solid media cultures, was highest in the starting CD34^+ ^cells and gradually decreased (Figure 1A). Cells displayed Mk morphological features including increased cell size and multilobated nuclei (Figure 1B) and some cells exhibited proplatelet formations (data not shown) as previously reported [9]. High-ploidy cells (≥8N), as detected by flow cytometry, started appearing on day 5 and reached a peak at day 12 with DNA content up to 16N (Figure 1C). A CD41a^+^AnnexinV^+^Propidium-iodide— apoptotic Mk cell population was detected by flow cytometry as early as day 5 and a significant increase in apoptosis was observed between days 9 and 12 (Figure 1D).

### A proposed set of comparative analyses to reliably identify Mk-related genes

**Figure 2:**
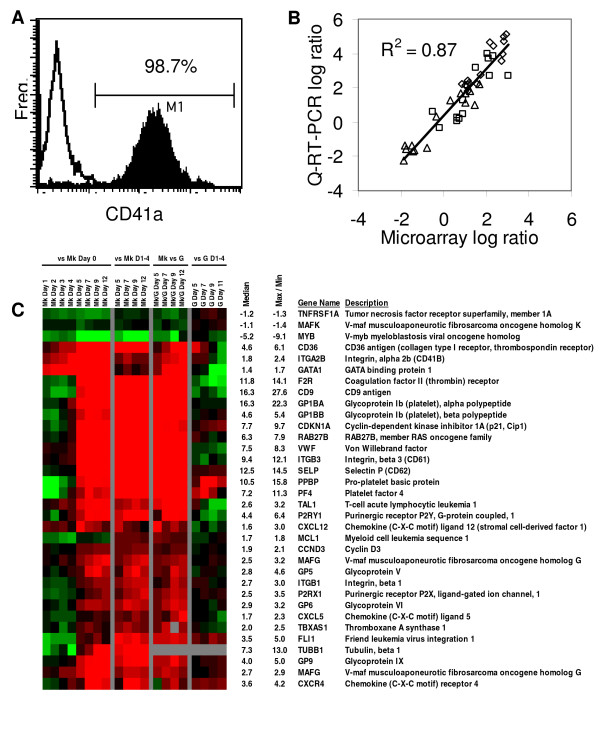
**Q-RT-PCR verification of selected microarray results and transcriptional analysis of Mk genes**. (A) For transcriptional analysis beginning on day 5, Mk culture samples were enriched for CD41a^+ ^cells by positive immunomagnetic selection. Flow cytometric analysis after selection revealed high purity. Data is shown for one representative sample point from one culture. Open line: isotype control; Filled line: cells after selection. (B) Q-RT-PCR validation of microarray results across multiple Mk culture samples. Microarray log expression ratios from Mk culture samples are compared to values obtained using Q-RT-PCR for each of 3 genes – BBC3 (squares), MAP3K5 (diamonds), and SIRT7 (triangles). (C) Expression profiles for 33 Mk genes that were differentially expressed with time in Mk cultures and/or between Mk and G cultures. Color denotes degree of differential expression (saturated red = 4-fold up-regulation, saturated green = 4-fold down-regulation, blank = unchanged, gray = no data available). The first block shows average expression ratios across the biological replicates (n = 3) for the designated samples as compared to day 0 CD34^+ ^cells; the second block shows expression ratios, averaged across the biological replicates (n = 3), as compared to the average expression from days 1 – 4; the third block shows average expression profiles as compared to equivalent-day G cells (day 12 Mk vs. day 11 for G cells) (n = 2); and the last block shows expression profiles of G cells with respect to average expression of G cells on days 1–4 (n = 2). The median ratio, along with the maximum (for up-regulated genes) or minimum (for down-regulated genes) ratio of Mk cells on days 5 – 12 (with respect to average expression of days 1–4) is provided (a negative value represents down-regulation).

The goal of this study is to combine ontological classifications with temporal DNA-microarray data in order to identify Mk-related genes involved in apoptosis. DNA-microarray-based transcriptional analysis was carried out using the mixed-cell population in Mk cytokine cocktail cultures from days 0 – 4 and, starting on day 5, using CD41a^+ ^cells enriched to above 97% by positive-selection using immunomagnetic beads (Figure 2A). As a control, transcriptional analysis was also performed on concurrent, isogenic granulocytic (G) cell cultures. These G cultures showed extensive G commitment as assessed by flow cytometry of G markers: on days 5 and 11, the G cell cultures were, respectively, 40% and 87% CD15^+^; 26% and 70% CD11b^+^; and 10% and 65% CD66b^+ ^(data not shown). Moreover, G cell cultures displayed rapid expansion, normal ploidy and, most importantly, no apoptosis detectable by Annexin V binding (data not shown). Crucial to the success of the gene identification and selection process is the combined use of three comparative, temporal gene expression analyses. (1) For the Mk cultures, the gene expression level at each time point is compared against that of CD34^+ ^cells from day 0 (n = 3). This comparison should identify genes regulated by the Mk-cytokine cocktail, including genes that are regulated as the CD34^+ ^cells shift from quiescence to cycling in response to initial cytokine stimulation. (2) We also compared the gene expression level in selected CD41a^+ ^cells (days 5–12; n = 3) against the average gene expression level in cells at days 1–4. This comparison should identify differentially expressed genes in committed Mk cells compared to the mixed population of days 1–4, which are more actively cycling and also express genes of other hematopoietic and tissue lineages. (3) We compared the average gene expression of selected CD41a^+ ^cells (days 5–12) against equivalent-day cells from concurrent, isogenic G cell cultures (n = 2). Due to the lack of apoptosis in the G cell cultures, this comparison is especially suitable for identification of Mk-related apoptosis genes. (4) Finally, we compared the gene expression levels in later days (starting on day 5) of G-culture cells compared to the average expression in days 1–4 (n = 2). This comparison should identify genes with differential expression patterns in differentiating G cells and, as we show below, adds further discrimination capability.

Of the approximately 18,000 genes probed by the arrays, 3918 were found to be differentially expressed in at least one of the aforementioned comparisons (see complete list in Additional File 1). The three biological replicate cultures showed highly reproducible gene expression patterns (see Additional File 2). This biological reproducibility is consistent with previous studies from our laboratory [21]. Thus, for simplicity and ease of presentation, we have averaged the gene expression data across cultures for presentation in the figures. Real-time quantitative reverse-transcription polymerase chain reaction (Q-RT-PCR) analysis was used to validate the microarray results of three differentially expressed genes, including two apoptosis-related genes discussed later (*BBC3*, *MAP3K5*; Figure 2B). As we have previously reported [20,21,23], the data from the Agilent microarrays strongly correlated with the Q-RT-PCR results, although Q-RT-PCR data generally show larger fold-changes compared to microarray analysis.

### Method assessment and the transcriptional program of core Mk genes

In order to assess the validity of the proposed strategy, we first aimed to apply it to a better defined and more general set of megakaryocytic genes. Because of the limited number of genes annotated under the ontological term "Megakaryocyte differentiation", we curated a list of 57 Mk genes based on existing literature [4,24-31] that are represented on the Agilent Human 1A(v2) array by a total of 64 probes (see Additional File 3). This set of genes and/or the proteins encoded by these genes have been established as associated with megakaryopoiesis through a variety of studies employing primary and immortalized, human and murine cells. However, their temporal transcriptional programs in primary human cells remain largely unknown. In addition to testing our proposed GO-driven method for identifying Mk-associated genes, our study aimed to also establish the temporal programs of these core Mk genes in primary human Mk cell cultures. We expected that several, but not all, of these genes would be transcriptionally regulated, but, for most of them, we had no means to anticipate which are transcriptionally regulated or what the strength of their transcriptional regulation might be based on their role or importance. Among these 57 curated genes, 33 genes (represented by 34 probes) were differentially expressed (Figure 2C). The four comparative analyses demonstrate a large repertoire of temporal expression patterns that cannot be uniquely captured by any single analysis or without temporal analysis of gene expression. For example the critical Mk transcription factor gene *GATA1 *is dramatically up-regulated by the early time-points of Mk culture, remains unchanged after day 5, and is down-regulated after day 7 in the G cultures. This is consistent with GATA1's role in both early and intermediate stages of Mk differentiation [32,33]. Like *GATA-1*, some Mk-related genes (*ITGA2B *(*CD41b*), *CD36*, *MAFG*) are expressed higher in both mixed and CD41a^+^-cell-enriched Mk-culture cells compared to day 0 CD34^+ ^cells, while many others (e.g. *PF4*, *ITGB3 *(*CD61*), *vWF*, and *SELP *(*CD62p*)) are expressed higher only in the selected CD41a^+ ^cells. Significantly, to the best of our knowledge, these temporal expression patterns, although they have not all been previously detailed in primary human cells, are consistent with prior literature and validate this approach to gene selection. As expected, many Mk- and platelet-specific transcripts were highly expressed in selected CD41a^+ ^cells. This included genes encoding a variety of integrins and receptors (*ITGA2B*, *ITGB3*, *GP5*, *GP6*, *GP9*, *CD36*, and *CD9*), cell cycle regulators (*CCND3*, *CDKN1A *(*p21*)), platelet granule proteins (*PPBP*, *SELP*, and *CXCL5*), and transcription factors (*GATA1*, *TAL1*, *MAFG*, *FLI1*). Finally, it is interesting to note that a few Mk-related genes (*PPBP*, *PF4*, *CD36*, *FLI1*), although expressed significantly higher in Mk cells compared to G cells, are transiently up-regulated in intermediate granulopoiesis (Figure 2C), as well.

In summary, the combination of four comparative gene expression approaches led to the identification of 58% (33/57) of the literature-based Mk-related genes – a significant enrichment over the 21% differential expression among all genes, despite the fact that not all of these 57 genes would be expected to be transcriptionally regulated. More importantly, this analysis identified most of the known transcriptionally-regulated Mk genes with the exception of some genes regulated by the late-Mk transcription factor NF-E2, such as myosin heavy polypeptide 9 (*MYH9*) and 3-β-hydroxy-steroid-dehydrogenase. In the Discussion, we also show that our method identifies most of the findings from a recent non-temporal transcriptional analysis of Mk colonies derived from cord-blood CD34^+ ^cells [34]. We next applied this strategy for the identification of apoptosis-related genes associated with Mk differentiation.

### The apoptosis transcriptome

**Figure 3:**
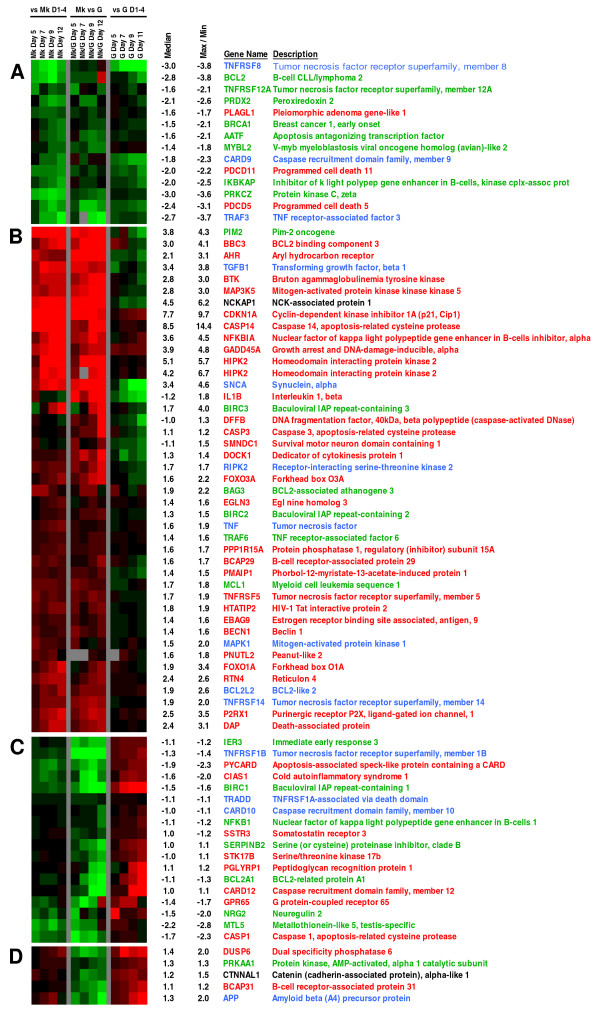
**Differentially expressed apoptosis-related genes in Mk cells**. Expression profiles of genes related to apoptosis (per Gene Ontology) that were differentially expressed temporally in Mk cultures and/or between Mk and G cultures. (A – D) Genes were divided into four groups according to their distinct expression patterns based on hierarchical clustering using the Euclidian distance metric. Color denotes degree of differential expression (saturated red = 4-fold up-regulation, saturated green = 4-fold down-regulation, blank = unchanged, gray = no data available). The first block shows expression ratios, averaged across the biological replicates (n = 3), with respect to the average expression from days 1 – 4.; the second block shows average expression profiles as compared to equivalent-day G cells (day 12 Mk vs. day 11 for G cells) (n = 2); and the last block shows expression profiles of G cells as compared to average expression of G cells on days 1–4 (n = 2). The median ratio, along with the maximum (for up-regulated genes) or minimum (for down-regulated genes) ratio of Mk cells on days 5 – 12 (with respect to average expression of days 1–4) is provided (a negative value represents down-regulation). Pro-apoptotic genes names and descriptions are highlighted in red, anti-apoptotic genes are highlighted green and genes with both pro- and anti-apoptosis roles are highlighted blue. Genes with unknown functions in apoptosis are in black.

Apoptosis is an integral part of terminal Mk differentiation and is required for proplatelet formation and platelet release [11,35,36]. Although many established molecular events in apoptosis are post-transcriptional and most cells constitutively express both pro- and anti-apoptotic components of the cell death machinery, it is nevertheless known that some of the apoptotic machinery is transcriptionally regulated [37]. Thus, we examined whether GO classification [22], combined with our microarray-based transcriptional data, can be used to identify Mk-related apoptotic genes. Using GO classification, we generated a list of 220 genes related to general and hematopoietic apoptosis that are represented on the Agilent Human1A(v2) array. We then employed the same comparative analyses used in Figure 2C. However, we show only the last three analyses (Figure 3A-D) because the comparison to day 0 CD34^+ ^cells is uninformative for apoptosis-related genes since initial cytokine treatment is likely to cause expression changes in apoptosis-related genes that are unrelated to Mk differentiation (false-positives) and terminal Mk apoptosis is not detected until day 5 (Figure 1D). As discussed above, the isogenic G cultures displayed no detectable apoptosis and cell viability remained above 93% (data not shown). Using these comparisons, we identified a list of 79 differentially-expressed apoptosis-related genes. Hierarchical clustering revealed distinct expression patterns for these 79 genes and allowed us to divide them into four clusters: (A) down-regulated after day 5 in both Mk and G cells compared to early days, but expressed lower in Mk than in G cells (Figure 3A); (B) up-regulated in Mk cells compared to both unselected early cells and G cells (Figure 3B); (C) down-regulated in Mk, but up-regulated in G cells after day 5 compared to early days (Figure 3C); and (D) up-regulated in both Mk and G cells after day 5 compared to early days, but expressed lower in Mk than in G cells (Figure 3D).

### Anti-apoptotic genes related to NF-κB signaling are down-regulated in both Mk and G cultures

Cluster A includes 14 genes that were down-regulated in both Mk and G cells but were more extensively down-regulated in Mk cells (Figure 3A), including 8 genes that encode anti-apoptotic proteins and 3 that encode pro-apoptotic proteins. The fact that this group has more anti-apoptotic genes (labelled with green text in Figure 3A) and that all showed faster or greater down-regulation in Mk cells correlates with the observation that apoptosis is only observed in the Mk cultures. BCL2 is a major anti-apoptotic regulator and over-expression of BCL2 results in decreased apoptosis and a significant reduction in platelet numbers [11,13]. Several genes in this cluster are associated with NF-κB signaling. These include *PDCD11*, which is a positive regulator for NF-κB signaling [38], and *IKBKAP*, which can bind NF-κB-inducing kinase (NIK) and IKKs and assemble them into an active kinase complex [39]. *TNFRSF12A *promotes survival via NF-κB activation and *Bcl-x*_*L*_/*Bcl-w *expression [40]. This provides preliminary evidence that NF-κB signaling is reduced during terminal Mk differentiation – an issue that is pursued further below.

### The kinetics of NF-κB activity and total versus phosphorylated NFkBIA levels suggest an important role for the NF-κB program in megakaryocytic apoptosis and differentiation

**Figure 4:**
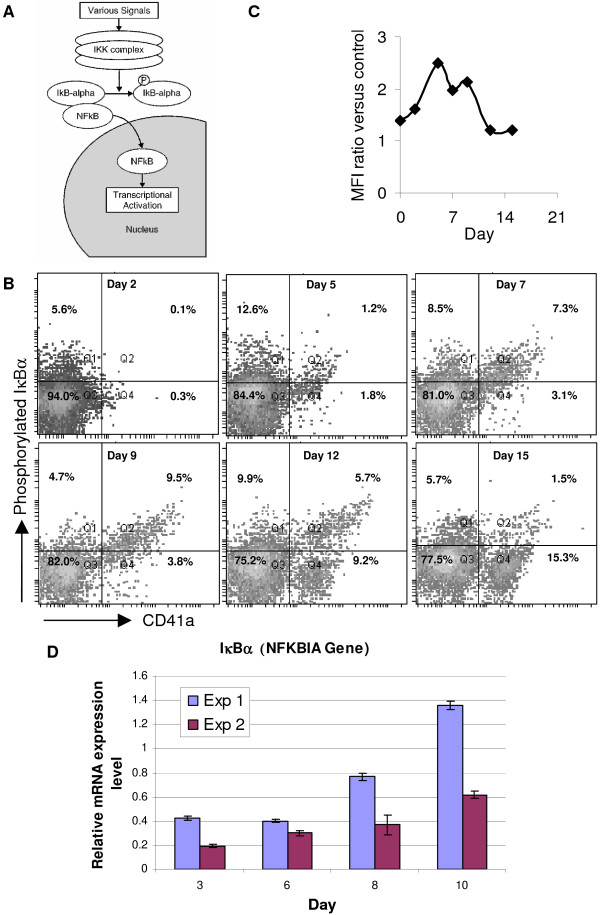
**NF-κB signaling increased during early Mk differentiation and decreased during terminal differentiation**. (A) Cartoon depicting simplified NF-κB signaling pathway. 'P' denotes phosphorylated form of protein. (B) IκBα phosphorylation in Mk cytokine-cocktail-stimulated cultures was examined by flow cytometry. Cells were stained with anti-CD41a-FITC and rabbit anti-phospho-IκBα (Ser32) primary antibody followed by APC-conjugated goat anti-rabbit secondary antibody. Results are from one representative experiment (n = 2). (C) Kinetics of NF-κB p65 phosphorylation (Ser529) in CD41a^+ ^cells from Mk cytokine-cocktail-stimulated cultures was measured by flow cytometry. Data shown is geometric mean fluorescence intensity of anti-phospho-p65-antibody stained samples relative to cells stained with isotype-matched control antibodies. Results are from one representative experiment (n = 2). (D) Q-RT-PCR analysis of IκBα mRNA expression in primary human CD34^+ ^cells stimulated with 100 ng/mL Tpo.

Our microarray data show that the genes encoding various units of NF-κB complex were either down-regulated (*REL*, *RELB*) or not differentially expressed (*NFKB1*, *NFKB2*, *RELA*) in Mk cell cultures. In contrast, *NFKBIA *mRNA, which encodes the α-subunit of NF-κB inhibitor IκB, was highly up-regulated in Mk cells (Figure 3B). Because of the aforementioned expression changes among NF-κB pathway members, we hypothesized that NF-κB signaling decreased during terminal Mk differentiation and apoptosis and sought to provide a first assessment of this hypothesis. The NF-κB transcription factor complex, which is an important regulator of cell proliferation and survival [41], is sequestered in the cytoplasm bound by members of the IκB family of inhibitors (predominantly IκBα; Figure 4A). NF-κB activation, which allows the translocation of NF-κB to the nucleus, requires dissociation of the inhibitor IκB (Figure 4A). This is accomplished, upon a suitable stimulus, by phosphorylation and subsequent ubiquitination and degradation of IκB (Figure 4A). Thus, IκB phosphorylation is a good indicator of NF-κB activity [42,43]. To assess this, we examined the IκBα phosphorylation in Mk cells by flow cytometry. Phosphorylated IκBα was detected in the majority of CD41a^+ ^cells during early differentiation but decreased after day 9 (Figure 4B). To further strengthen these initial investigations on the hypothesized role of NF-κB, we also examined phosphorylation of p65 (the *RELA *gene product) – an established indicator of optimal NF-κB activity [44] – in Mk cells by flow cytometry. The amount of phosphorylated p65 in Mk cells increased during the early days, reached a peak at day 5, and then steadily decreased (Figure 4C). We also examined the mRNA expression pattern of IκBα by Q-RT-PCR and found that IκBα expression correlated with Mk differention state (Figure 4D). In both experiments, IκBα expression was high (ca. 3–3.5 higher than the day 10 mRNA level; data not shown) on day 0 (uncultured CD34^+ ^cells), decreased to low levels during the early stages of megakaryopoiesis (day 3) and then gradually increased as the cells became more mature Mk cells (day 10), thus confirming our microarray data. The experiments shown (Figure 4D) used primary human CD34^+ ^cells from two different donors with somewhat different rates of Mk commitment; experiment 2 showed reduced Mk commitment based on CD41 expression levels (data not shown), and had lower levels of IκBα expression compared to experiment 1 which displayed a stronger Mk commitment.

**Figure 5:**
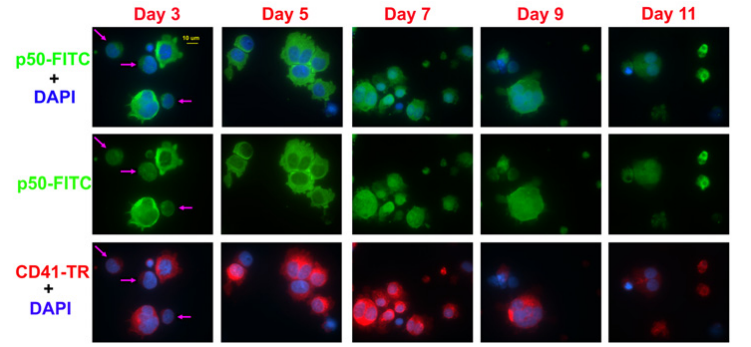
**Immunofluorescence microscopy analysis of p50-NLS**. Human mobilized peripheral blood CD34^+ ^cells were cultured in Tpo and harvested on the indicated day of culture to analyze the expression and localization of the p50 nuclear localization sequence (NLS) via immunofluorescence microscopy. Cells were co-stained with DAPI (blue) and antibodies   against p50-NLS (green) and CD41a (red). All images were captured using a 63X oil immersion objective.

**Figure 6:**
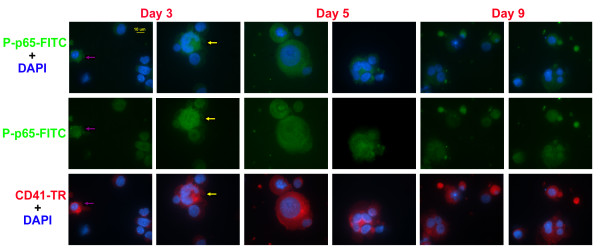
**Immunofluorescence microscopy analysis of phospho-Ser536-p65**. Human mobilized peripheral blood CD34^+ ^cells were cultured in Tpo and harvested on the indicated day of culture to analyze the expression and localization of phosphorylated p65 (Ser536) via immuno-fluorescence microscopy. Cells were co-stained with DAPI (blue) and antibodies   against phosphor-Ser536-p65 (green) and CD41a (red). All images were captured using a 63X oil immersion objective.

To further test for changes in NF-κB activity during terminal Mk differentiation, we examined the sub-cellular localization of two activated components of the NF-κB complex by staining with antibodies raised to the nuclear localization sequence (NLS) of p50 or serine-phosphorylated-p65 (Ser536). The expression of p50 was dim and cytoplasmic in CD34^+ ^hematopoietic stem cells (data not shown), but early Mk differentiation (days 3 and 5) brought about a pronounced cytoplasmic accumulation of p50 (Figure 5). The effect was significantly weaker in CD41-, non-Mk-committed cells found in culture (pink arrows). Upon further Mk differentiation, p50 remained cytoplasmic, but also translocated to the nucleus and reached the peak of its expression on day 7. The level of p50 decreased during late Mk differentiation (days 9 and 11) and p50 expression was low in the nucleus and the cytoplasm. Together, these data indicate a dynamic temporal expression pattern for p50 during Mk differentiation, suggesting that NF-κB signaling is active in early Mks, but decreases as the cells mature. In parallel experiments, the expression of Ser536-phosphorylated p65 was very low in CD34^+ ^hematopoietic stem cells (data not shown), but intensified soon after Tpo-induced differentiation (Figure 6, day 3), which is consistent with the flow cytometry data previously presented (Fig. 4C). While phosphorylated p65 was cytoplasmic in megakaryoblasts (purple arrow), it was found in both the nucleus and cytoplasm in more mature Mks, as identified by characteristic Mk morphology (yellow arrow). Concurrent with this sub-cellular shift in p65 localization, Ser536-phosphorylated p65 levels increased as Mk cells matured and reached a peak on day 5. Phosphorylated p65 (Ser536) levels then declined during further Mk differentiation (days 7, 9, and 11). We show a representative image from day 9, but similar images were commonly found for days 7 and 11. Taken together, these data support our hypothesis that NF-κB signaling decreases during late Mk maturation and apoptosis, but as discussed below, further investigations will be necessary to delineate its role and impact on megakaryopoiesis.

### The up-regulated apoptosis-related genes are enriched in pro-apoptotic components including *GADD45A*, *MAP3K5*, various p53 targets, and components of the TNF signaling pathway

**Figure 7:**
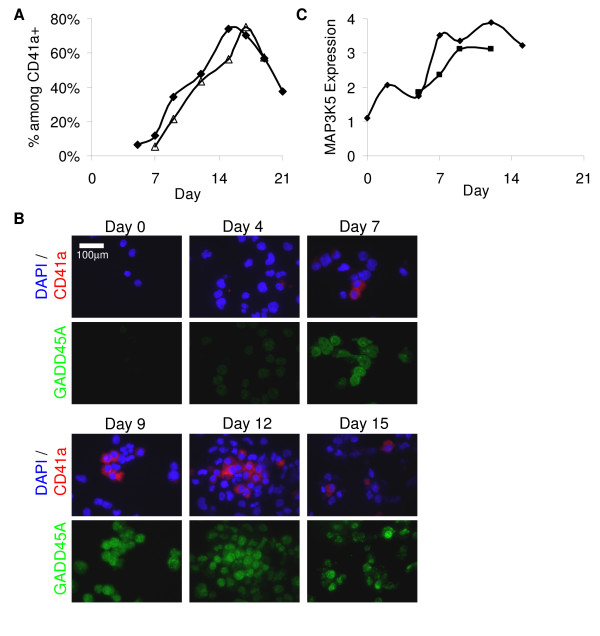
**Analysis of apoptotic protein expression in Mk cultures**. (A) Kinetics of active (cleaved) caspase-3 (triangles) correlates strongly with apoptosis as measured by AnnexinV/PI method (diamond) in cytokine-cocktail-stimulated Mk culture as assayed by flow cytometry. Data is from one representative experiment (n = 3). (B) Cells from cytokine-cocktail-stimulated Mk cultures were co-stained with DAPI (blue), and antibodies against CD41a (red) and GADD45A (green) from day 0 to day 15. Images shown are representative fields (n ≥ 3 per slide). Results are from one representative experiment (n = 3). Image acquired with 20× objective. (C) MAP3K5 (ASK1) protein expression (diamonds) in cytokine-cocktail-stimulated Mk cultures as represented by geometric mean fluorescence intensity of anti-MAP3K5-antibody stained samples relative to cells stained with isotype-matched control antibodies (representative data from n = 2 experiments). Protein expression correlates with average MAP3K5 (ASK1) mRNA expression (squares) in CD41a^+ ^cells as measured by microarray compared to average expression in cytokine-cocktail-stimulated Mk cultures from day 1 – 4 (n = 3). MAP3K5 and GADD45A exhibited similar protein expression patterns in cultures stimulated with Tpo alone (data not shown; n = 2).

The difference in progression of apoptosis between the Mk and G cultures suggests there should be an enrichment of pro-apoptotic genes in cluster B. These 42 genes (Figure 3B) are highly enriched, as predicted, in pro-apoptotic genes. Namely, they include 27 genes that encode for pro-apoptotic proteins (shown in red text), only 6 genes that encode for anti-apoptotic proteins (green text), and the balance play amphi-apoptotic or unknown roles (blue text). Overall, this cluster identifies a unique set of genes that were up-regulated during, and may be responsible for, the progression of Mk apoptosis. We note that this cluster contains one of the few known players in Mk apoptosis, namely the effector caspase-3, which is activated upon cleavage by caspase-9 during Mk apoptosis [11]. The caspase-3 transcript level was only slightly up-regulated among Mk cells, thus suggesting that our analysis can capture even subtle, yet significant, transcriptional effects. To assess the protein activity of caspase-3, flow-cytometric analysis was carried out and revealed that, after day 7, the active-form caspase-3 significantly increased and correlated well with progression of apoptosis in the Mk culture (Figure 7A).

Most of the other genes present in this cluster have not been previously associated with Mk apoptosis and provide new insights into the transcriptional control of Mk apoptosis. Among members of the extrinsic pathway, components of the TNF-signaling cascade (*BIRC3 *(*cIAP2*), *BIRC2 *(*cIAP1*), *TNFRSF5*, and *TRAF6*), but not the Fas-signaling pathway, were found to be up-regulated. There was significantly more activity among the components of the intrinsic apoptosis pathway. Several members of the Bcl-2 family were found in this cluster, including both pro-apoptotic and anti-apoptotic regulators. *BBC3 *(*PUMA*) and *PMAIP1 *(*NOXA*) are pro-apoptotic members of the Bcl-2 family under p53 control in response to DNA damage [45,46]. Three other p53-regulated apoptotic genes – *GADD45A*, *PPP1R15A*, and *CDKN1A *(*p21*) – were included in this cluster. The involvement of p53 in terminal Mk differentiation is further supported by our unpublished observations that RNA-interference-mediated knock-down of p53 in the megakaryoblastic CHRF cell line leads to an increase in viability and enhanced endomitosis upon phorbol-ester-induced terminal differentiation (manuscript in preparation). We used immunofluorescence microscopy to examine if GADD45A was expressed at the protein level in Mk-cocktail cultures (Figure 7B). GADD45A expression in CD41a^+ ^cells was low at day 4, increased from day 7 to day 15, and was higher in CD41a^+ ^cells than in CD41a^- ^cells. It was expressed in both the nucleus and cytoplasm with increasing nuclear localization, particularly among CD41a^+ ^cells on days 12 and 15. Similar expression patterns were seen in Tpo-only Mk cultures (data not shown).

Also in this cluster was *MAP3K5*, also known as Apoptosis Signal-Regulating Kinase 1, which encodes a kinase that participates in the ERK and JNK signaling pathways [47] and contributes to apoptosis in various cell types [48-51]. We evaluated MAP3K5 protein expression by intracellular flow cytometry and found that, among CD41a^+ ^Mk cells from cytokine cocktail cultures, it was markedly higher from day 7 to day 15 than on earlier days (Figure 7C). MAP3K5 was also detected in Mk cells cultured with Tpo alone, with a similar increasing trend (data not shown). The correlation of protein and mRNA expression in parallel with the onset of apoptosis, suggests that pro-apoptotic signaling through MAP3K5 is involved in promoting Mk apoptosis.

### The last two clusters (up-regulated in G but lower in Mk cultures) contain genes related to either apoptosis or hematopoietic differentiation

Cluster C includes 18 apoptosis-related genes that were up-regulated in G cells and either down-regulated or unchanged in Mk cells (Figure 3C). Of these, 8 encode pro-apoptotic proteins and 7 encode anti-apoptotic proteins. This group may contain genes necessary for G differentiation, but not necessary for Mk differentiation, but may also contain anti-apoptotic genes affecting Mk apoptosis or preventing G apoptosis. For example, the Bcl-2 family member BCL2A1 can reduce the release of pro-apoptotic cytochrome C from mitochondria and block caspase activation, and is a direct transcriptional target of NF-κB in response to inflammatory mediators. This group may also contain genes which, though generally classified as pro-apoptotic, are not necessarily associated with apoptosis during hematopoietic differentiation. Specifically, the caspase-1 (*CASP1*) and *CARD12 *gene products facilitate the proteolytic activation of the IL-1β precursor protein, which is necessary for myeloid differentiation [52,53]. Down-regulation of IL-1β activators CASP1 and CARD12 in Mk cells and their up-regulation in G cells would suggest a lineage-specific fine-tuning of IL-1β-dependent autocrine regulation.

Finally, the small cluster D includes five genes that were up-regulated in both Mk and G cells after day 5 compared to early days, but were expressed higher in G cells (Figure 3D). These are genes that might play a role in Mk cells and also are likely to be involved in differentiation, rather than apoptosis, in G cells. *DUSP6 *encodes for a cytoplasmic dual-specificity protein phosphatase that inactivates ERK1/2 and has been shown to induce apoptosis in endothelial cells and pancreatic cancer cells [54-56]. The *BCAP31 *gene product can be cleaved by caspase-8 and subsequently promotes cytochrome C release [57,58]. It may also play a role in regulating intracellular trafficking of CD11b/CD18 in neutrophils [59], and thus is important in G differentiation. Amyloid beta (A4) precursor protein (APP) has been found in both α-granules of platelets and phagocytic granules of neutrophils [60,61]. PRKAA1 is expressed in neutrophils and its activation inhibits the respiratory burst [62].

### Expression pattern of apoptosis-related genes is independent of Mk cytokine mixture

In independent experiments, we examined the global gene expression profile of mobilized peripheral blood CD34^+ ^cell cultures stimulated with Tpo alone, as opposed to the three cytokine cocktail used in the present study [20]. Culture with Tpo alone yields higher CD41a^+ ^cell purity, lower total expansion, and, most importantly, enhances terminal maturation including polyploidization and proplatelet formation compared to the cytokine cocktail. Comparison of the gene expression patterns for the Mk-related and apoptosis-related genes discussed in this manuscript showed nearly perfect agreement between Tpo-only and Mk cytokine cocktail cultures (data not shown). Furthermore, the protein-level microscopy and flow cytometry studies were repeated on Tpo-only cultures yielding concurring results (data not shown). This suggests that the observations and conclusions from this study hold more broadly and are not dependent on a specific culture system.

## Discussion

The goals of this study were to demonstrate the importance of high-quality temporal transcriptional data and of targeted comparative analyses of such data for identifying genes underlying, at the transcriptional level, an important phenotype such as Mk apoptosis. It is clear that pursuing the functional role of each and every gene identified by such an approach is a tall and time-consuming objective. It was therefore important to develop various means for validating our approach with the goal of providing a high degree of confidence in our findings. Nevertheless, we sought to validate some selected candidate genes at the protein and activity level as a first assessment of their functional role.

NF-κB signaling is involved in Tpo and SDF-1 signaling in proliferating Mk cells [63,64]. Furthermore, NF-κB is spontaneously activated in Mk cells from idiopathic myelofibrosis (IMF) patients and plays a role in regulating *TGF-β1 *expression, but not cytokine-withdrawal-induced apoptosis, in IMF-patient-derived Mk cells [64]. However, its role in late Mk differentiation is not clear. We have, for the first time, shown evidence in primary human Mk cells that NF-κB signaling decreases during late Mk differentiation. This suggests that decreased NF-κB signaling may be required for Mk cells to undergo apoptosis. Zhang [65] came to the same conclusion based on their study of a Tpo-responsive murine Mk cell line. In agreement with this, Leger and colleagues [66] have recently shown that NF-κB signaling is inhibited in the human HEL cell line when induced to undergo Mk differentiation. In order to firmly establish the role of NF-κB in terminal megakaryopoiesis, future studies will be needed to investigate the effect of forced NF-κB signaling perturbations on Mk culture outcomes, as well as on elucidating signaling upstream and downstream of NF-κB.

In our examination of Mk-related apoptosis genes, we detected mRNA and protein-level up-regulation of GADD45A. GADD45A can induce apoptosis by mediating the translocation of Bim to the mitochondria [51]. In addition to apoptosis, GADD45A mediates G2/M arrest in a p53-dependent manner involving the depletion of nuclear cyclin B [67]. This cell cycle arrest has been associated with increased translocation of GADD45A to the nucleus, as was observed late in Mk cultures (Figure 5B). The importance of this observation is underscored by the numerous investigations into the cell cycle-dependent decrease in cyclin B/Cdc2 activity associated with Mk endomitosis (reviewed by Ravid [28]) and by our recent report of increased p53-DNA binding activity during Mk differentiation of the megakaryoblastic CHRF-288-11 cell line [21]. While our data strongly suggest that GADD45A plays a role in either Mk apoptosis or endomitosis or both, further study will be necessary to clearly establish its functional relevance.

To date, most microarray studies of Mk differentiation have used a single time point or disease state comparisons, rather than temporal gene expression analysis. Two recent reports have explored progressions of Mk differentiation using gene expression microarrays. One report discussed the transcriptional profiling of culture-derived murine Mk cells that had been sorted into discrete developmental stages by light-scattering properties and CD41 expression using flow cytometry [68]. Separately, Raslova *et al *separated culture-derived human Mk cells by ploidy class and subjected them to transcriptional analysis using the Agilent microarray platform [69]. In the present study, we compared the time-course expression profile of differentiating Mk and G cells to identify genes with potential roles in terminal Mk apoptosis. Key to this approach was the use of a reference design in our microarray analysis that allowed us to simultaneously compare between time-points from different cultures. The validity of the proposed approach is based largely on the ability to identify correctly some previously known property of the genes. One example is confirmation of the expectation, based on our method, that the genes of Figure 3A are indeed mostly anti-apoptotic genes. A second example is that our method identifies correctly most of what has been previously known about the transcriptional regulation of core Mk genes (Figure 2C). As an additional test, we pursued the later issue further as follows. Balduini [34] established a set of "Mk-core" genes by contrasting cord-blood-derived Mk colonies with isogenic erythroid colonies and uncultured progenitor cells. We examined the expression of these "Mk-core" genes in our cultures and found that of the 55 Mk-core genes represented on our arrays by 58 probes, 41 genes (42 probes) were differentially expressed (see Additional File 4). More significantly, all differentially expressed genes were up-regulated in the Mk-cultures. While some Mk-core genes were also expressed in the G cultures, only one (*Annexin A3*) was expressed higher in the G cells than the Mk cells. These assessments, together with the preliminary protein-level and activity assessments of a few select genes (Figures 4 and 5) suggest that the large number of new genes identified by our analysis as potentially associated with Mk apoptosis indeed has great biological significance.

Significantly, our data (Figure 2C) provide a comprehensive temporal transcriptional analysis of core Mk genes from which we have identified Mk genes that are strongly or weakly up-regulated and genes which are not apparently transcriptionally regulated. Furthermore, these data on the temporal expression of the core Mk genes can be co-clustered and correlated with the identified Mk-related apoptosis genes (Figure 3A) in an effort to identify possible regulatory relationships, an effort much beyond the scope of this article.

## Conclusion

In this study, we have both demonstrated the utility of a large-scale, dynamic, comparative genomics approach to improving our understanding of apoptosis within the context of Mk differentiation. We have identified and preliminarily verified the transient activation of the NF-κB pathway during Mk differentiation. Additional candidate Mk apoptosis genes were identified including *BBC3*, *MAP3K5 *(*ASK1*), and *GADD45A*. In addition, these data provide new information about the temporal regulation of known Mk genes. The numerous leads from this work will be the seeds for future work pursuing in-depth functional studies of individual candidate genes.

## Methods

### Hematopoietic cultures

Mk and G cell cultures were initiated with fresh or frozen G-CSF-mobilized human peripheral blood CD34^+ ^cells from normal donors (AllCells; Berkeley, CA) and cultured as described [10,20,70]. For Mk cultures, cells were cultured in X-VIVO 20 serum-free medium (BioWhittaker; Walkersville, MD) supplemented with either a cocktail of Mk-inducing cytokines (50 ng/mL Tpo (Genentech; South San Francisco, CA or Peprotech; Rocky Hills, NJ), 50 ng/mL Flt-3 ligand (PeproTech), and 5 ng/mL IL-3 (R&D Systems; Minneapolis, MN)) or Tpo alone (100 ng/mL). Tpo was added to the cultures every 5 days. Mk cultures were seeded at 70,000 cells/mL and cultured for up to 21 days at 37°C in a fully-humidified, 5% CO_2 _environment. G cultures were performed in serum-containing human long-term medium supplemented with (R&D Systems), 10 ng/mL IL-6 (Peprotech), 10 ng/mL G-CSF (Amgen; Thousand Oaks, CA), 50 ng/mL stem cell factor, and 10 ng/mL IL-3 (both R&D Systems) at 37°C in a fully-humidified environment containing 5% CO_2 _and 5% O_2_.

### Mk colony-forming assay

Mk colony-forming assays were performed as previously described [10] using MegaCult™-C media (Stem Cell Technologies; Vancouver, BC, Canada). All colonies containing more than three CD41a^+ ^cells were scored as Mk colony-forming units (CFU-Mk).

### Flow cytometry

Surface marker expression was assessed as described [10] using phycoerythrin (PE)-labeled anti-CD41a and fluorescein isothiocyanate (FITC)-labelled anti-CD34 monoclonal antibodies (Coulter; Fullerton, CA; and Becton Dickinson, respectively). Propidium iodide (PI) was used to exclude dead cells. Apoptosis was assessed using Annexin V-FITC/PI in combination with PE-labeled-CD41a as described [10]. Ploidy was assayed as described using anti-CD41a-FITC/PI staining [10]. Polyploid Mk cells were defined as CD41a^+ ^cells with 8N or higher DNA content. For intracellular detection of active-caspase-3, MAP3K5 (ASK1) and phosphorylated IκBα, cells were first stained with anti-CD41a-FITC and then fixed, permeabilized, and stained as previously described [71]. Rabbit-anti-human phosphorylated IκBα antibody was from Cell Signaling Technology (Danvers, MA), and other primary antibodies were from Santa Cruz Biotechnology (Santa Cruz, CA). Secondary antibodies were from Jackson ImmunoReseach (West Grove, PA). The NF-κB activity was measured with anti-NF-κB p65 phospho-Ser529-specific antibody with PERM III buffer following the manufacturer's instructions (Becton Dickinson). Flow cytometry data were acquired on a FACScan or LSRII flow cytometer (Becton Dickinson).

### Microarray experiments and data analysis

Cell samples were flash frozen in liquid nitrogen at day 0 for CD34^+ ^cells; days 1, 2, 3, 4, 5, 7, 9, and 12 for Mk cell cultures; and days 1, 2, 3, 4, 5, 7, 9, and 11 for G cell cultures. Starting from day 5, Mk cell culture samples were enriched by CD41a^+ ^selection using MiniMACS MS columns (Miltenyi Biotec; Auburn, CA) prior to freezing. Briefly, cells from the Mk culture were washed with phosphate-buffered-saline (PBS) containing 2 mM EDTA and 0.5% bovine serum albumin, labelled with anti-CD41a-PE antibody (Coulter) and separated with anti-PE microbeads (Miltenyi Biotec). After day 12, RNA yields and integrity in the Mk cultures were too low to continue with array analysis (data not shown), likely as a result of significant apoptosis and low cell viability. Total RNA was isolated using the RNA Isolation Mini-kit (Agilent Technologies; Wilmington, DE). Sample RNA and Universal Reference RNA (Stratagene; La Jolla, CA) were linearly amplified and labelled using the Low RNA Input Fluorescent Linear Amplification Kit following the manufacturer's instructions and hybridized as described [20]. Microarray slides were scanned with the Agilent Microarray Scanner (G2565BA). Approximately one third of the individual microarrays were replicated and the correlation coefficient between these technical replicates was 0.83 – 0.97.

Feature intensity statistics were extracted using Agilent's Feature Extraction software (G2567AA, version 7.2). For further analysis, spots with signal intensity near or below background were discarded as previously described [72], then duplicate spots were averaged, data were normalized, and the normalized ratios for technical replicates were averaged [73]. For Mk vs. G comparison, the Mk/G value was first calculated for each experiment and then averaged. Raw and normalized data were deposited in the **Gene Expression Omnibus **[77] (Mk cells: GSE3839; G cells: GSE5917). All subsequent data analysis was performed using the MultiExperiment Viewer 3.0 (MeV; Institute for Genomic Research, Rockville, MD) [74]. Differentially expressed genes were identified using the statistical analysis of microarrays (SAM; for Mk temporal comparisons) or analysis of variance (ANOVA; for comparison of Mk to G cultures) as implemented in MeV with a false discovery rate <5% and p < 0.05, respectively. Gene Ontology annotations, as curated by European Bioinformatics Institute, were retrieved from the Gene Ontology Consortium website [78]. Hierarchical clustering was performed with the Euclidian distance metric.

### Quantitative (Q)-RT-PCR

cDNA was obtained from total RNA samples using the High-Capacity cDNA Archive Kit and Q-RT-PCR was performed with Assays-on-Demand kits (Applied Biosystems; Foster City, CA) as described [20]. The amount of mRNA for each sample was normalized using the average of two housekeeping genes (Glucuronidase-β and 18S). The use of GUSB and 18S genes as housekeeping genes has been previously tested in our lab [20,21,75]. The primer codes were as follows: *GUSB *(Hs99999908_m1), *18S *rRNA (Hs99999901_s1), *BBC3 *(Hs00248075_m1), *MAP3K5 *(Hs01039896_m1), *SIRT7 *(Hs00213029_m1), *NFKBIA *(Hs00153283_m1; normalized only to *RPLP0*: Hs99999902_m1).

### Microscopy

Cells from Mk cultures were deposited onto microscope slides by cytocentrifugation (Shandon; Pittsburgh, PA). For Wright-Giemsa staining, cells were fixed in methanol, stained with Camco QuickStain II (Camco, Fort Lauderdale, FL) and washed with PBS and distilled water. Immunofluorescence microscopy for p50 and GADD45A was performed as previously described [76]. Briefly, cells were fixed in 4% paraformaldehyde (Electron Microscopy Sciences, Ft. Washington, PA) in PBS, permeabilized in 0.3% Triton X-100 in PBS, blocked with 10% normal goat serum in PBS/2% bovine serum albumin, then stained with rabbit anti-human p50 or rabbit anti-human GADD45A (Santa Cruz Biotechnology, Santa Cruz, CA) and mouse anti-human CD41a (Beckman-Coulter, Fullerton, CA) primary antibodies or rabbit anti-human IgG and mouse anti-human IgG (both from Santa Cruz Biotechnology) primary antibodies followed by FITC-conjugated goat anti-rabbit IgG and Texas-Red-conjugated goat anti-mouse IgG secondary antibodies (Jackson Immunoresearch). Finally, samples were mounted with Prolong Gold anti-fade reagent with DAPI (Molecular Probes, Eugene, OR). The cells that were stained with the antibody against phosphorylated p65 and their isotype controls were fixed with 2.5% paraformaldehyde in PBS, permeabilized in 0.3% Triton-X in PBS containing 5% normal goat serum then stained with rabbit anti-human phosphorylated p65 (Cell Signaling, Danvers, MA) and mouse anti-human CD41 (Beckman-Coulter) or rabbit anti-human IgG and mouse anti-human IgG (both from Santa Cruz Biotechnology) as recommended by Cell Signaling. Finally these samples were stained with secondary antibodies and DAPI as described above. All microscopy was performed with 63× (N.A. = 1.32 in oil) or 20× (N.A. = 0.40 CS) objectives on a Leica Model DMIRE2 inverted microscope (Wetzlar, Germany) outfitted with a QImaging Retiga EXi CCD camera (Burnaby, BC, Canada) and captured into OpenLab imaging software (Improvision; Lexington, MA).

## List of abbreviations

Mk: megakaryocytic

G: Granulocytic

GO: Gene Ontology

IMF: idiopathic myelofibrosis

PI: propidium iodide

PBS: phosphate-buffered-saline

FITC: fluorescein isothiocyanate

PE: phycoerythrin

Q-RT-PCR: quantitative reverse-transcription polymerase chain reaction

## Authors' contributions

CC and LH carried out the culture experiments and performed the microarray experiments. CC, PF, and CP analyzed the microarray data. CC and PF wrote the manuscript. PA and MW performed immunofluorescence microscopy and Q-RT-PCR, respectively. CC, PF, WM, and EP conceived of, designed, and coordinated the study. All authors read and approved the final manuscript.

## Supplementary Material

Additional file 1 Complete differentially expressed gene list. Listing of all genes that were differentially expressed either over the time-course of Mk cytokine-cocktail-stimulated mobilized peripheral blood CD34^+ ^cell cultures or between equivalent time points of G and Mk cultures. Click here for file

Additional file 2 Reproducibility of expression profiles in biological replicate Mk cultures. Data shown for all genes that were at least 2-fold up- or down-regulated versus day 0 in at least two samples across all 25 total sampling points. Color denotes degree of differential expression (saturated red = 4-fold up-regulation, saturated green = 4-fold down-regulation). Click here for file

Additional file 3 Listing of Mk-related genes based on literature review. The complete Mk-associated gene list is presented with common gene name, description, and appropriate references from the review literature. Click here for file

Additional file 4 Expression profiles of the differentially expressed members of the Mk-core gene set (as reported by Balduini). These genes were differentially expressed in our cultures as described in the text. Color denotes degree of differential expression (saturated red = 4-fold up-regulation, saturated green = 4-fold down-regulation). The first block shows average expression ratios across the biological replicates (n = 3) for the designated samples with respect to day 0 CD34^+ ^cells; the second block shows expression ratios, averaged across the biological replicates (n = 3), with respect to the average expression from days 1 – 4.; the third block shows average expression profiles with respect to equivalent-day G cells (day 12 Mk vs. day 11 for G cells) (n = 2); and the last block shows expression profiles of G cells with respect to average expression of G cells on days 1–4 (n = 2). Click here for file
